# Oxygen tension regulates the miRNA profile and bioactivity of exosomes released from extravillous trophoblast cells – Liquid biopsies for monitoring complications of pregnancy

**DOI:** 10.1371/journal.pone.0174514

**Published:** 2017-03-28

**Authors:** Grace Truong, Dominic Guanzon, Vyjayanthi Kinhal, Omar Elfeky, Andrew Lai, Sherri Longo, Zarin Nuzhat, Carlos Palma, Katherin Scholz-Romero, Ramkumar Menon, Ben W. Mol, Gregory E. Rice, Carlos Salomon

**Affiliations:** 1 Exosome Biology Laboratory, Centre for Clinical Diagnostics, University of Queensland Centre for Clinical Research, Royal Brisbane and Women’s Hospital, The University of Queensland, Brisbane, Queensland, Australia; 2 Maternal-Fetal Medicine, Department of Obstetrics and Gynecology, Ochsner Clinic Foundation, New Orleans, United States of America; 3 Division of Maternal-Fetal Medicine & Perinatal Research, Department of Obstetrics & Gynecology, The University of Texas Medical Branch at Galveston, Galveston, Texas, United States of America; 4 Robinson Research Institute, Discipline of Obstetrics and Gynaecology, School of Medicine, University of Adelaide, North Adelaide, Australia; 5 Department of Clinical Biochemistry and Immunology, Faculty of Pharmacy, University of Concepción, Concepción, Chile; Colorado State University, UNITED STATES

## Abstract

Our understanding of how cells communicate has undergone a paradigm shift since the recent recognition of the role of exosomes in intercellular signaling. In this study, we investigated whether oxygen tension alters the exosome release and miRNA profile from extravillous trophoblast (EVT) cells, modifying their bioactivity on endothelial cells (EC). Furthermore, we have established the exosomal miRNA profile at early gestation in women who develop pre-eclampsia (PE) and spontaneous preterm birth (SPTB). HTR-8/SVneo cells were used as an EVT model. The effect of oxygen tension (i.e. 8% and 1% oxygen) on exosome release was quantified using nanocrystals (Qdot^®^) coupled to CD63 by fluorescence NTA. A real-time, live-cell imaging system (Incucyte^™^) was used to establish the effect of exosomes on EC. Plasma samples were obtained at early gestation (<18 weeks) and classified according to pregnancy outcomes. An Illumina TrueSeq Small RNA kit was used to construct a small RNA library from exosomal RNA obtained from EVT and plasma samples. The number of exosomes was significantly higher in EVT cultured under 1% compared to 8% oxygen. In total, 741 miRNA were identified in exosomes from EVT. Bioinformatic analysis revealed that these miRNA were associated with cell migration and cytokine production. Interestingly, exosomes isolated from EVT cultured at 8% oxygen increased EC migration, whilst exosomes cultured at 1% oxygen decreased EC migration. These changes were inversely proportional to TNF-α released from EC. Finally, we have identified a set of unique miRNAs in exosomes from EVT cultured at 1% oxygen and exosomes isolated from the circulation of mothers at early gestation, who later developed PE and SPTB. We suggest that aberrant exosomal signalling by placental cells is a common aetiological factor in pregnancy complications characterised by incomplete SpA remodeling and is therefore a clinically relevant biomarker of pregnancy complications.

## Introduction

Remodelling of the uterine spiral arteries (SpA) during early pregnancy is requisite for establishing efficient materno-fetal nutrient and oxygen transfer. During the first half of pregnancy, ~100–150 SpAs are converted from high resistance low flow vessels with a diameter of ~200μm [1] to high flow low resistance vessels with a diameter of ~ 2 mm [2]. The initial processes invoved in remodeling are: vessel dilatation, vascular smooth muscle cell (VSMC) separation, endothelial cell (EC) swelling, infiltration of extravillous trophoblast (EVT) cells and fibrinoid deposition [3]. VSMCs migrate out of the vessels and/or undergo apoptosis. They are then replaced by EVT cells [4]. It has been proposed that an oxygen concentration gradient, established between the lumen of the uterus (~3% O_2_) and the myometrium (12% O_2_), drives EVT cell migration from the anchoring villi of the placenta into the decidua and myometrium. Intraluminal EVT cells occlude SpAs to maintain a low oxygen environment that is requisite for normal early placental and fetal development. Towards the end of the first trimester, low resistance, high capacity flow is achieved by the loss of intraluminal EVT plugs and the placental intravillous space is perfused with maternal blood, establishing effective materno-fetal exchange. Aberrant SpA remodelling has been implicated in the subsequent development of pregnancy complications, including preeclampsia, compromised fetal growth and spontaneous preterm birth [5] [6].

The mechanisms by which EVT affect resident cell phenotypes have yet to be clearly established. We propose that exosomal signaling between EVT, VSMCs and EC promotes SpA remodeling [7]. Consistent with this hypothesis, we have previously reported that exosomes are released from an EVT immortalised cell line (HTR8/SVneo) and promote the migration of vascular smooth muscle *in vitro*. To further elucidate the putative role of EVT exosomal signaling in SpA remodelling, the aim of this study was to establish the effects of oxygen tension on the release and miRNA content of EVT-derived exosomes, and the effect of these exosomes on EC phenotype.

In recent years, exosomal signaling has been identified as a novel and significant cell-to-cell communication pathway [8–10]. Exosomes are nanovesicles encapsulated by a phospholipid bilayer (40-120nm in diameter) that are exocytosed into biofluid compartments. They are packaged with distinct signaling molecules, including proteins, lipids, mRNAs, miRNAs and non-coding RNAs. Exosomes regulate the activity of both proximal and distal target cells, affecting translational activity, angiogenesis, proliferation, metabolism and apoptosis. They may be assembled and secreted in response to signals received from neighboring cells, from distant tissues or in response to local environmental factors (*e*.*g*. oxygen tension, glucose and free fatty acid concentration) [8, 11]. Previously, we have reported that exosomes are released from cytotrophoblast (CT) cells *in vitro*. Additionally, we have shown that placenta-derived exosomes *(i*.*e*. placental alkaline phosphatase positive, PLAP^+^, CD63^+^) are present in maternal plasma as early as 6 weeks of pregnancy. Furthermore, the release, protein content and bioactivity of CT-derived exosomes is regulated by glucose and oxygen concentration [12]. The aim of this study was to test the hypotheses that: *i*) the release, miRNA content and bioactivity of EVT-derived exosomes is regulated by oxygen tension; and *ii*) maternal plasma exosomal miRNAs are differentially expressed during early pregnancy in pre-symptomatic women who subsequently develop preeclampsia and spontaneous preterm birth (SPTB); conditions associated with incomplete SpA remodeling. We propose that aberrant exosomal signaling by placental cells is a common aetiological factor across a spectrum of pregnancy-associated complications characterized by placental dysfunction and defective remodeling of the uterine spiral arteries, and is therefore a clinically useful biomarker of complications of pregnancy.

## Materials and methods

### Cell culture

All experimental procedures were conducted within an ISO17025 accredited (National Association of Testing Authorities, Australia) research facility. All data was recorded within a 21 Code of Federal Regulation (CFR) part 11 compliant electronic laboratory notebook (Lab Archives, Carlsbad, CA 92008, USA). The HTR-8/SVneo cell line was kindly donated by Dr. Charles H. Graham (Queen's University, Ontario, Canada). HTR-8/SVneo was established by the transfection of trophoblast cells isolated from first trimester villous explants and containing a gene encoding the Simian virus 40 large T antigen to immortalize them [13]. HTR-8/SVneo is commonly used as an EVT model and HTR-8/SVneo authentication of human trophoblast cell line was performed with a STR DNA Profiling Analysis (S1 Fig). HTR-8/SVneo cells were maintained in phenol red-free RPMI 1640 medium supplemented with 10% heat-inactivated fetal bovine serum (FBS), 1% non-essential amino acids, 1 mM sodium pyruvate, 100 U/mL penicillin, and 100 mg/mL streptomycin. Cultures were maintained at 37°C and humidified under an atmosphere of 5% CO_2_-balanced N_2_ and either 8% or 1% O_2_ in an automated PROOX 110-scaled hypoxia chamber (BioSpherics^™^, Lacona, NY, USA). Cells were cultured in RPMI 1640 medium supplemented with 10% FBS-exosomes depleted for 48h before exosome isolation. Cells were sub-cultured with dissociation media, TrypLETM Express (Life technologies, USA). Cellular viability was determined by Trypan Blue exclusion solution and Countess^®^ Automated cell counter (Life Technologies, USA).

### Patients

A case control study design was used to establish pregnancy-associated changes in exosomal miRNA present in maternal plasma obtained from normal, preeclampsia and preterm birth pregnancies. Human plasma samples were obtained in accordance with the Declaration of Helsinki. This study was approved by the ethics committee of The University of Queensland (HREC/09/QRBW/14) and Ochsner Medical Foundation (USA). Women were recruited with informed written consent at the Ochsner Baptist Medical Center (New Orleans, USA). Samples were obtained before 20 weeks of gestation and classified according to the pregnancy outcomes in normal, PE or SPTB. PE was defined as new-onset hypertension [(BP) blood pressure ≥140/90mmHg on two separate occasions at least 6h apart or BP ≥160/110mmHg)] and proteinuria (>300mg/24h) after 20 weeks of gestation in previously normotensive women, according to the International Society for the Study of Hypertension in Pregnancy [14]. SPTB was defined as a birth before gestational week 37 + 0. The clinical characteristics of the patients included in this study are presented in Table 1.

**Table 1:** Clinical characteristics of patients.

Variables			
	Normal (n = 6)	PE (n = 6)	SPTB (n = 6)
Maternal age (years)	31 ± 2.9 (29–35)	32 ± 4.3 (28–33)	30 ± 5.4 (26–33)
Height (cm)	165 ± 0.6 (160–170)	159 ± 0.7 (155–170)	160 ± 0.7 (150–165)
Weight (Kg)	61 ± 5.3 (55–68)	63 ± 5.3 (55–70)	61 ± 4.6 (55–69)
BMI (Kg/m^2^)	23 ± 1.5 (20–26)	25 ± 1.3 (20–29)	24 ± 1.8 (20–27)
Systolic/diastolic blood pressure (mm Hg)	109/65 ± 6.22/9.12 (90-120/50-81)	155/69 ± 10/8* (>140/90)	111/67 ± 10/7 (90-120/50-80)
Gestational age at sample collection (weeks)	17 ± 3.5 (15–18)	16 ± 2.6 (15–18)	16 ± 2.8 (15–18)
Gestational age at delivery (weeks)	40 ± 1.2 (37–42)	38 ± 0.9 (37–39)	35 ± 1.1 (34–36)

Data are presented as mean ± SD (range). PE = preeclampsia; SPTB = spontaneous preterm birth.

*p<0.05 vs Normal and SPTB.

### Isolation of exosomes

Exosomes were isolated from EVT-conditioned medium (720 ml of cell-conditioned media obtained from ~600 x 10^6^ cells) or maternal plasma (1 ml) as previously described [12, 15–17]. Briefly, samples were centrifuged at 300 x g for 10 min, 2,000 x g for 10 min, and 12,000 x g for 20 min; and, 2,000 x g for 30 min, and 12,000 x g for 45 min to remove whole cells and debris for cell-conditioned media and plasma, respectively. The resultant supernatant fluid was passed through a 0.22 μm sterile filter (Steritop^™^, Millipore, Billerica, MA, USA) and then centrifuged at 100,000 x g for 120 min (Thermo Fisher Scientific Inc., Asheville, NC, USA, Sorvall, SureSpinTM 630/36, Tube angle: 900). The pellet was resuspended in PBS (500 μl, pH 7.4), layered on the top of a discontinuous iodixanol gradient containing 40% (w/v), 20% (w/v), 10% (w/v) and 5% (w/v) iodixanol solutions made by diluting a stock solution of OptiPrep^™^ (60% (w/v) aqueous iodixanol from Sigma-Aldrich) and centrifuged at 100,000 x g for 18 h. Twelve individual fractions were collected, diluted in PBS and centrifuged at 100,000 x g for 2 h. Finally, the pellet for each fraction was resuspended in 50 μl of PBS and stored at -80°C.

### Nanoparticle Tracking Analysis (NTA)

NTA measurements were performed using a NanoSight NS500 instrument (NanoSight NTA 3.0 Nanoparticle Tracking and Analysis Release Version Build 0064) as previously described [12, 18–20]. Each of the twelve fractions was diluted to 100 μg/ml protein and 5 videos were processed and analyzed. A minimum of 200 completed tracks per video were collected for each analyzed sample. NTA post-acquisition settings were optimized and kept constant between samples, and each video was then analyzed to give the mean, mode, and median particle size together with an estimated number of particles per mL plasma or per 10^6^ cells A spreadsheet (Excel, Microsoft Corp., Redmond, Washington) was automatically generated, recording the concentration of each particle size. 100 nm polystyrene latex microspheres (Malvern NTA 4088) were routinely analyzed to confirm instrument performance.

### Fluorescence NTA

Qdots (Qdot^®^ nanocrystals) were conjugated to anti-CD63 or IgG1 isotype control antibody (IgG1 sc-34665, Santa Cruz Biotechnology) with a SiteClick Qdot 605 Antibody Conjugation Kit (Life Technologies), executed according to the manufacturer’s instructions as previously described [21]. Exosomes were diluted in PBS and incubated with FcR blocking reagent (10 μl, 10 min at 4°C) (MACS Miltenyi Biotec), followed by incubation with anti-CD63-Qdot605 or IgG1-Qdot605 (10 μl, 1:100) for 30 min in the dark at room temperature. Samples (*i*.*e*. (i) exo-EVT alone, (ii) exo-EVT + IgG1-Qdot605 and (iii) exo-EVT + anti-CD63-Qdot605 and background controls (iv) FcR blocking reagent + IgG1-Qdot605 and (v) FcR blocking reagent + anti-CD63-Qdot605) were then diluted to 500 μL with PBS and analyzed using the NanoSight NS500 instrument and NTA software. For each sample, five 60 second videos were captured and analysed in fluorescence mode (*i*.*e*. camera level 9, shutter speed 11.25 ms and slider gain 250).

### Western blot analysis and transmission electron microscopy

Exosomal proteins separated by polyacrylamide gel electrophoresis were transferred onto polyvinylidene difluoride membranes using the Trans-Blot^®^ Turbo^™^ Transfer System (BioRad Laboratories). After transfer, the blot was blocked with Odyssey^®^ Blocking Buffer (OBB) at room temperature for 1hr. The blocking buffer was removed and anti-TSG101 or anti-HLA-G antibodies (Abcam) diluted 1:1000 in OBB was added and incubated overnight at 4°C. The blot was then washed for 5 mins in Tris-buffered saline supplemented with 0.1% v/v Tween 20 (TBST). This was repeated twice. Anti-rabbit conjugated with DyLight 800 secondary antibody diluted 1:15,000 in OBB was added. This was followed by washes of 5 mins each of TBST and an additional wash of TBS for 5 mins to remove any residual Tween-20. The blot was scanned using a LI-COR^®^ Biosciences Odyssey^®^ Infrared Imaging System and data quantified using Image Studio software version 4.0. For electron microscopy analysis, exosome pellets were fixed in 3% (w/v) glutaraldehyde and analyzed under an FEI Tecnai 12 transmission electron microscope (FEI^™^, Hillsboro, Oregon, USA).

### Endothelial cell isolation

Primary human umbilical vein endothelial cells isolated from normal pregnancies were used as the EC model. Cells were isolated by enzymatic digestion as previously described [12] and used as an *in vitro* model to determine the effect of exosome bioactivity on EC.

### Internalization of exosomes

The internalization of exosomes by EC was assessed as previously described [19] using fluorescently labelled (PKH67 green, Sigma-Aldrich) exosomes.

### Effect of exosomes on EC migration

EC were cultured in media 199 supplemented with 0.2% FBS-exosome free in a 96-well ImageLock Microplate (ESSEN BioScience Inc, Ann Arbor, Michigan, USA) according to the manufacturer’s instructions for 18–24 h. During experiments, EC were incubated in the presence (treatment: 100 μg exosomal protein per mL of incubation medium) or absence (control) of EVT-derived exosomes obtained from EVT cells cultured under 1% or 8% O_2_ for up to 48 h (n = 6). Experiments involving EC were performed under an atmosphere of 8% O_2_ to mimic the physiological conditions. The concentration used in this study was based upon exosome dose-response curves from our previously published studies [15, 22, 23]. Cell migration was assessed using a scratch assay format. A scratch was made on confluent monolayers using a 96-pin WoundMaker^™^ (ESSEN BioScience Inc, Ann Arbor, Michigan, USA). Wound images were automatically acquired and registered by the IncuCyte^™^ software system. CellPlayer^™^ 96-Well Invasion Assay software was used to fully automate data collection. Data are presented as the Relative Wound Density (RWD, Eizen, v1.0 algorithm). RWD is a representation of the spatial cell density in the wound area relative to the spatial cell density outside of the wound area at every time point (time-curve). Migration assays were performed in the presence of Mitomycin C (100 ng/ml) to minimize any confounding effects of cell proliferation. The rate of wound closure was compared using the half-maximal stimulatory time (ST_50_) and area under the time course curve (AUC).

### Effect of exosomes on TNFα release and expression from EC

To determine the effect of exosomes on cytokine release from target cells, exosomes were isolated from the cell-conditioned media of EVT cells incubated under either 8% or 1% O_2_. Exosomes (100 μg protein/mL equivalent to 5 x 10^8^ vesicles per mL) were then incubated with primary human umbilical vein endothelial cells (HUVECS) in medium containing 5 mM d-glucose under an atmosphere of 8% O_2_ to mimic the physiological conditions for 24 h. TNFα release, defined as the accumulation of immuno-reactive cytokine in cell-conditioned medium (soluble and particle-associated), was quantified using a protein solution array assay as previously described [12]. Post-treatment EC-conditioned media was centrifuged at 100,000g x 2 h. The concentration of TNFα was quantified in the supernatant (*e*.*g*. soluble TNFα) and pellet (particle-associated TNFα). Data are expressed as pg of cytokine/10^3^ cells/24h. As a further control, exosomes were subjected to heat inactivation (30 min at 65°C) or sonication for 30 min (Elma, Elmasonic s15h, ultrasonic frequency 37 khz) before incubation with EC. Total RNA from HUVECs in the absence or in the presence of exosomes from EVT cells was extracted according to standard protocols and converted to first strand cDNA using the RT^2^ First Strand Kit. The template was added to an instrument-specific, ready-to-use RT^2^ SYBR Green QPCR Master Mix. The primer pair for rat TNFα was a RT^2^ qPCR Primer Assay (Qiagen). The reactions were performed in triplicate using the BIO-RAD iQ^™^5 Multicolor Real-Time PCR Detection System (USA) with the following conditions: 94°C for 3 min, 35 amplification cycles of 94°C for 45 s, 55°C for 30 s and 72°C for 30 s, 72°C for 10 min, 12°C for ∞ min. For each experimental group, 3 samples were run in triplicate. Relative quantification of gene expression was calculated by 2^−ΔΔCt^ and are shown as folds of increased messenger RNA transcription after normalization over the control values (GAPDH).

### Exosomal RNA isolation

Exosomal RNA was extracted using the RNeasy Mini Kit 50 (Qiagen, Australia) and TRIzol LS Reagent (Life Technologies, Australia). The manufacturers’ protocol was followed, with slight modifications in accordance with a protocol used previously [24]. A spectrophotometer (SPECTROstar Nano Microplate Reader, BMG LABTECH) was used to quantify RNA concentration. Following a cleanliness check and blank measurement using RNase-free water, 2 uL of sample was pipetted on to each microdrop well on an LVis plate. RNA concentration was measured using MARS Data Analysis microplate reader software.

### Next generation sequencing

Sequencing libraries were generated using the TruSeq^®^ SmallRNA Library Prep Kit, according to the manufacturer’s instructions. A total of 1 μg RNA or 100 to 300 ng of exosomal RNA was used as input for library preparation. These RNA samples were barcoded by ligation with unique adaptor sequences to allow pooling of samples in groups of 24. Subsequently, these ligated samples were reverse transcribed, PCR amplified and size selected using gel electrophoresis. Finally, the DNA libraries were eluted from the gel pieces overnight in 200 μL nuclease free H_2_0. The elution containing the pooled DNA library was further processed for cluster generation and sequencing using NextSeq 500 High Output kit 75 cycles and Illumina NextSeq 500 sequencing platform, respectively.

### Identification of miRNAs in raw sequencing data

Initially, raw FASTQ files were processed to remove barcode and adaptor sequences. Subsequently, this file was analysed using the miRDeep2 program to identify known miRNAs [25]. The miRDeep2 algorithm requires a genomic index and miRNA database to perform analysis. The human genome (version 19) pre-built index was obtained from the bowtie website (http://bowtie-bio.sourceforge.net/index.shtml). The miRNA reference database (version 20) was obtained from the miRBase website (http://www.mirbase.org/) [26]. Sequencing data have been deposited in the GEO database (GSE93020).

### Bioinformatic analysis

Gene target identification for miRNAs was performed using the CyTargetLinker application in Cytoscape. Firstly, candidate miRNAs identified from the sequencing data was imported into Cytoscape. A total of 3 miRNA gene target databases (MicroCosm, miRTarBase and TargetScan) were allocated to Cytoscape for analysis. Subsequently, gene targets were filtered to select for those identified to be targeted by the same miRNA within at least two databases. From this selection, genes shown to be regulated by at least two miRNAs were extracted and subjected to gene ontology analysis. Gene ontology analysis was performed on miRNA gene targets, using the BiNGO application in Cytoscape. Statistics were performed using a hypergeometric test, and corrected for multiple testing using the Benjamini and Hochberg procedure.

### Statistical analysis

The effects of oxygen tension on the release of exosomes from EVT are presented as the number of exosomes released from 10^6^ cells/48h (mean ± SE, *n* = 6 independent isolations from 300 million cells each). The effect of exosomes on cytokine release from EC is presented as pg cytokine/10^3^ cells/24 h (mean ± SE, n = 6 independent experiments from 3 placentae). The effects of oxygen tension on the release and bioactivity of exosomes (cell migration and cytokine release) were statistically evaluated using ANOVA. Statistical differences between groups were identified by *post hoc* analyses using Bonferroni’s tests to compare each treatment to the control group where the data distribution approximates normality.

Differential expression and statistical analysis of sequencing data was performed by the DESeq2 package in R [27]. This package uses a generalised linear model to perform differential expression. Statistical analysis and significance were calculated using a Wald test and adjusted for multiple testing using the Benjamini and Hochberg procedure. Adjusted p-value <0.01 = *, <0.001 = **, <0.0001 = *** was designated to be statistically significant. Heatmaps and graphs were produced using the “gplots” and “ggplot2” packages respectively in R.

## Results

### Effect of oxygen tension on exosomes released from EVT

**Fig 1:**
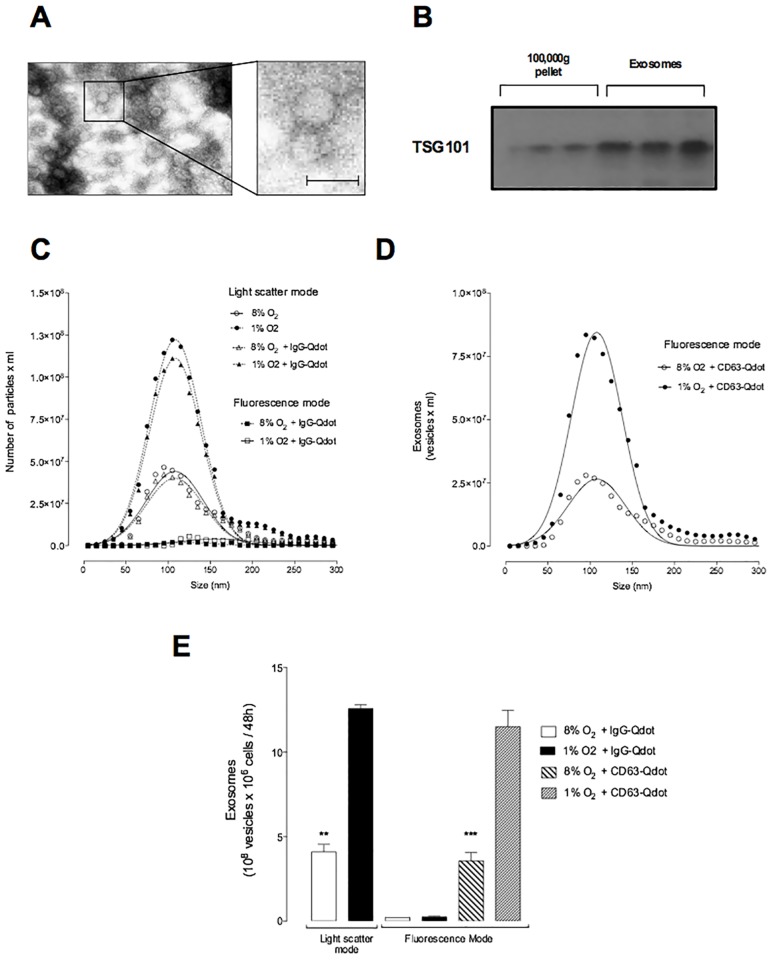
Effect of low oxygen tension on the release of exosomes. The effect of oxygen tension (8% and 1% O_2_) on the release of exosomes from EVT cells was quantified using NanoSight in light scatter and fluorescence mode. (A) Electron micrograph of exosomes isolated by ultracentrifuge and purified with a buoyant density gradient (pooled exosomal pellet density from 1.13 to 1.19 g/ml). (B) enrich of TSG101 protein abudance. (C) Size distribution of exosomes (pool enrich fractions) using exosomes or exosomes-Qdot-IgG. (D) Size distribution of exosomes (pool enrich fractions) using samples incubated with Qdot-CD63. (E) Quantification of from C and D. In A, Scale bar 100 nm. In B and C, none of the experiments performed were significantly different in Normal vs. Low oxygen tension. In E, data is presented as the number of exosomes released x 10^8^/ 10^6^ cells/ 48h. Values are mean ± SEM (n = 6 independent isolations from 300 x 10^6^ cells each). In E, **p<0.01; ***p<0.001.

Extracellular vesicles were isolated from EVT-conditioned media and enriched using isopycnic centrifugation (S2 Fig). Exosome density (1.13 to 1.19 g/ml) and size distribution (108 ± 15 nm), measured using anti-CD63-functionalized nanocrystals (Quantum dots, CD63-Qdot), were not significantly affected by oxygen tension (*i*.*e*. 8% and 1% O_2_, Table 2). Exosome enriched fractions (fractions 5 to 8) contained ~100 nm diameter vesicles identified by electron microscopy (Fig 1A), and were enriched for TSG101 (as assessed by Western blot analysis, (Fig 1B).

**Table 2:** Effect of oxygen tension on the size distribution of exosomes.

		Mean ± SD (nm)			Mode ± SD (nm)		
	Density (g/ml)	8% O2	1% O2	p value	8% O2	1% O2	p value
Fraction 1	1.078	196 ± 101	129 ± 123	0.3268	190 ± 70	130 ± 60	0.1420
Fraction 2	1.088	144 ± 54	153 ± 29	0.7266	220 ± 55	140 ± 60	0.0368*
Fraction 3	1.095	166 ± 89	173 ± 47	0.8681	175 ± 55	220 ± 59	0.2017
Fraction 4	1.116	135 ± 45	130 ± 29	0.8237	150 ± 50	120 ± 33	0.2481
^&^Fraction 5	1.125	125 ± 59	110 ± 37	0.6093	120 ± 35	100 ± 25	0.2813
^&^Fraction 6	1.145	110 ± 25	115 ± 20	0.7101	100 ± 25	98 ± 25	0.8925
^&^Fraction 7	1.166	109 ± 30	108 ± 26	0.9520	105 ± 22	104 ± 20	0.9360
^&^Fraction 8	1.178	100 ± 37	105 ± 46	0.8398	110 ± 20	85 ± 22	0.0664
Fraction 9	1.193	144 ± 54	150 ± 39	0.8298	130 ± 44	200 ± 55	0.0352*
Fraction 10	1.201	154 ± 64	133 ± 56	0.5587	125 ± 30	160 ± 50	0.1722
Fraction 11	1.225	134 ± 44	153 ± 29	0.3979	140 ± 33	170 ± 40	0.1868
Fraction 12	1.245	123 ± 31	157 ± 44	0.1528	150 ± 35	110 ± 50	0.1395

*p<0.05 between 8% and 1% O_2_.

^&^exosomes fractions.

The effect of oxygen tension on the release of exosomes from EVT cells was established using the NTA under both light scatter and fluorescent modes (Fig 1C–1E). The total number of vesicles was quantified under light scatter mode and CD63 postive (a late endosomal exosome marker) vesicles were quantified using CD63-Qdot. Oxygen tension had no effect on the size distribution of exosomes released from EVT in either light scatter mode or fluorescent mode (*i*.*e*. CD63^+^), showing that the exosome-Qdot binding did not affect vesicle characteristics. In fluorescent mode and in the absence of Qdots, no particles were identified (Fig 1C and 1E). A similar number of particles were identified in light scatter mode in the absence and in the presence of Qdot-IgG (~90% *i*.*e*. nonspecific binding of ~10%). Interestingly, the percentage of CD63^+^ vesicles in the total vesicle population (defined as total vesicles in light scatter mode) was ~92 ± 5% and ~89 ± 6% for 8% and 1% O_2_, respectively (Fig 1C), indicating that the majority of the isolated vesicles were CD63^+^. These results establish that the release of exosomes from EVTs is significantly higher (~3-fold) at 1% O_2_ than 8% O_2_ (Fig 1D and 1E).

### Effect of oxygen tension on EVT exosomal miRNA content

**Fig 2:**
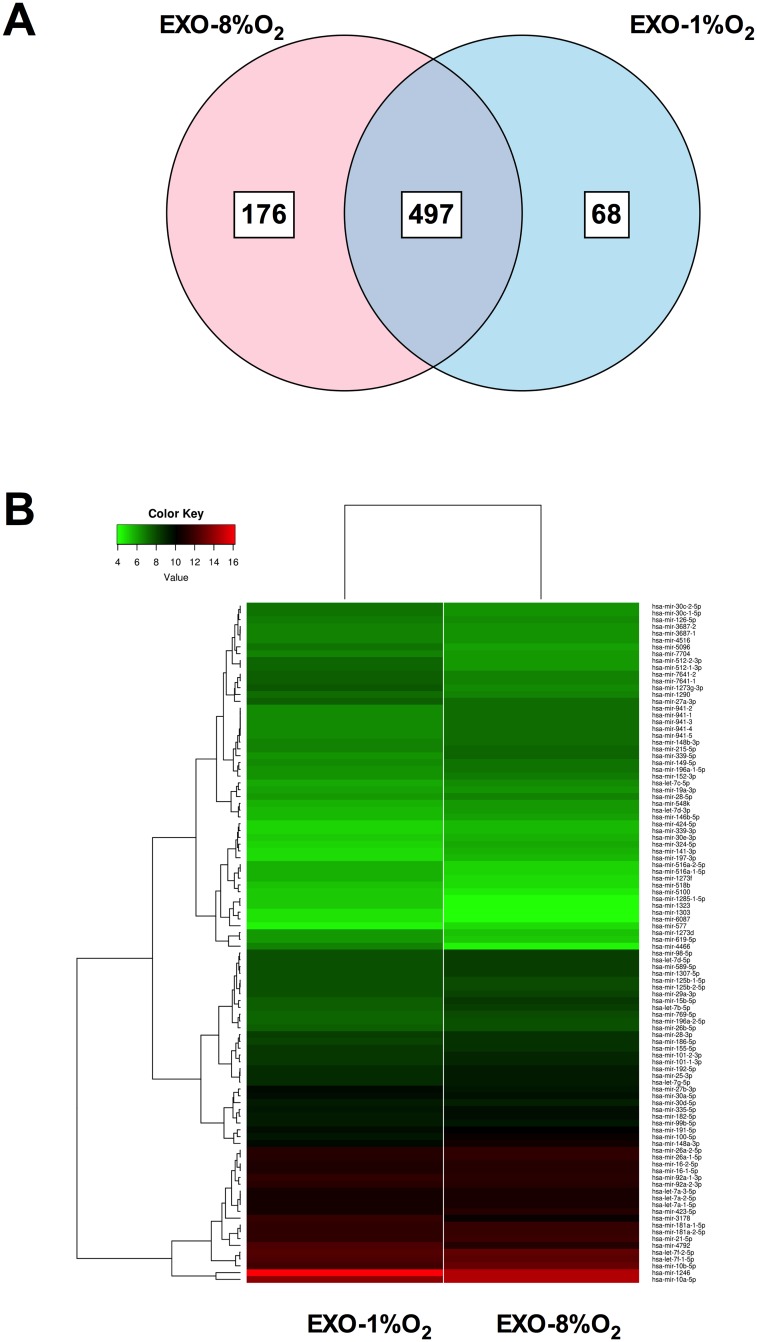
miRNA profile in EVT-derived exosomes under low oxygen tension. (A) The Venn diagram depicts the distribution of common and unique miRNA identified in exosomes released from EVT exposed to 1% and 8% oxygen. (B) Top 100 abundant miRNAs classified by normalised counts (see Methods) identified in exosomes isolated from EVT cells cultured under 1% or 8% oxygen. Hierarchical clustering of deep sequencing data was performed across exosomal miRNA samples. Normalisation of the reads was performed by DESeq2 using factors derived from the media ratio method.

Analysis identified 741 unique miRNA (-3p and -5p) species in exosomes derived from EVT cells. The average across all three samples was calculated to obtain a representative profile of biological diversity between samples. 176 and 68 unique miRNAs were identified in exosomes isolated from EVT cultured at 8% and 1% O_2_, respectively (Fig 2A). Hierarchical clustering of deep sequencing data is presented in Fig 2B, revealing the classification of miRNAs into two large clusters. This clustering shows that the majority of the top 100 abundance miRNAs for SpA remodeling have low expression within exosomes isolated from EVT grown at 1% O_2_ as opposed to 8% O_2_.

Statistical analysis of the common miRNAs identified within exosomes from EVT cultured at 8% and 1% O_2_ that were uniquely expressed in exosomal samples was conducted by applying a filter of p-value < 0.01 and at least a 2-fold change in either direction using Ingenuity pathway analysis. In total 10 miRNAs were up-regulated (miR-339-5p, miR-16-5p, miR-10a-5p, miR-935, miR-197-3p, let-7a-5p, miR-324-5p, miR-15b-3p, miR-100-5p, miR-365-3p) and 10 miRNAs were down-regulated (miR-675-5p, miR-1246, miR-293-5p, miR-3178, miR-4792, miR-548o-3p, miR-1273g-3p, miR-762, miR-3182, miR-523-3p) in exosomes isolated from EVT cultured at 1% O_2_ compared to those isolated from a normal condition of 8% O_2_.

**Fig 3:**
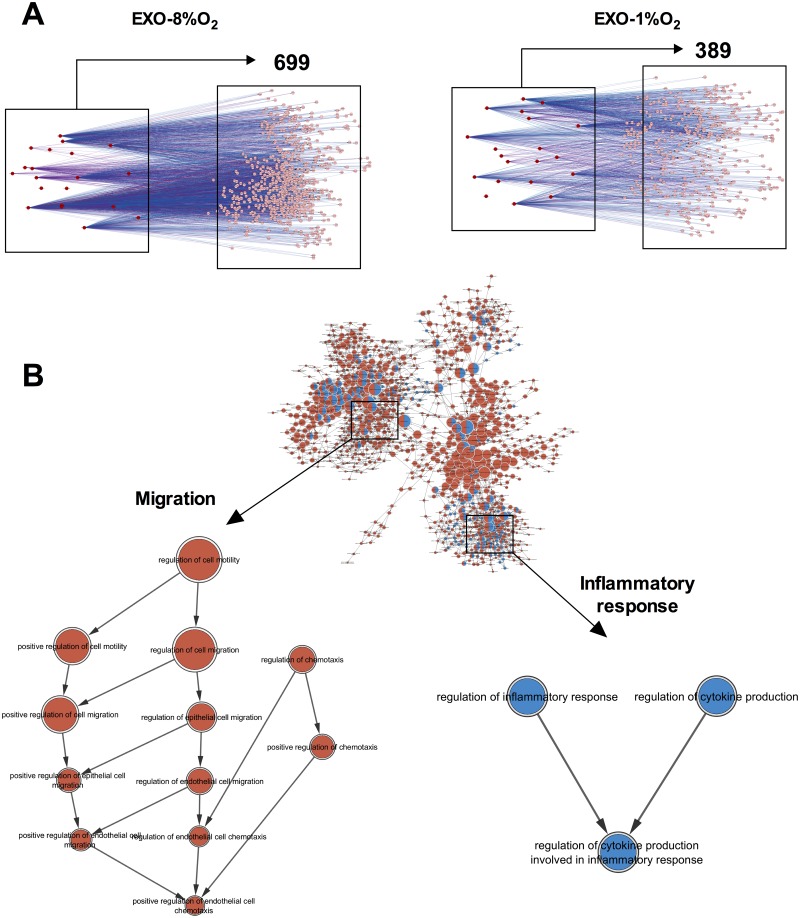
Target gene prediction of miRNAs identified in EVT-derived exosomes. (A) Gene target identification using CyTargetLinker was performed on the top 20 miRNAs in exosomes from EVT cultured at 8% or 1% oxygen. The genes were identified to be regulated by at least two of our candidate miRNAs, and are detected within at least two miRNA-gene target databases. (B) Top: Gene Ontology analysis using BiNGO was performed on all genes and displayed as a network. Bottom: Gene ontology pathway extracted from exosomes obtained from EVT at 8% and 1% oxygen showing the “migration” and “inflanmmatory response” gene ontology term, respectivetly.

Gene target identification using CyTargetLinker was performed on the top 20 abundant and unique miRNAs for exosomes isolated from EVT cultured at 8% and 1% oxygen. A total of 699 and 389 miRNA regulated genes for exosomes derived from EVT cultured at 8% and 1% respectively was detected within at least two miRNA-gene target databases (Fig 3A). Gene ontology analysis using BiNGO was performed on the 699 and 389 genes identified from exosomes isolated from EVT cultured at 8% and 1% oxygen respectively, and displayed as a merged gene ontology network (Fig 3B). The merged network contained a total of 1108 gene ontology terms, where exosomes from EVT cultured at 8% (red) and 1% (blue) had 1042 and 204 gene ontology terms, respectively (Fig 3B). Exosomes isolated from EVT cultured at 8% oxygen had genes associated with cell migration, while exosomes isolated from EVT cultured at 1% had genes linked with the regulation of cytokine production and the inflammatory response.

### EVT-derived exosomes bioactivity on EC

**Fig 4:**
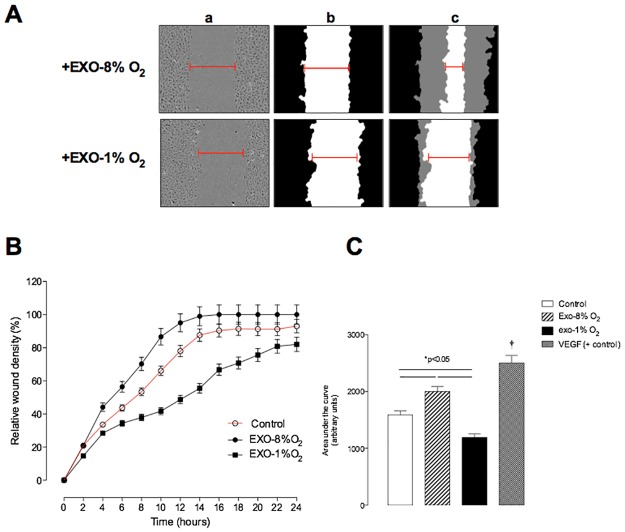
Effect of exosomes on endothelial cell migration. Endothelial cells (EC) were grown to confluence in complete media, wounds were made using 96 well WoundMaker and culture in absence or presence of exosomes (100 μg protein/ml equivalent to 5 x 10^8^ vesicles per ml) obtained from EVT cultured under 8% or 1% O_2_. (A) a, EC image immediately after wounding; b, Graphical representation showing the calculation of initial wound width; c, Graphical representation of cell migration at the midpoint of the experiment. (B) The time course of the effect of exosomes on EC. (C) Area under curves from data in B. Data represents n = 12 well for each point (6 different experiment in duplicate) Values are mean ± SEM. ^†^p<0.05 versus all values.

**Fig 5:**
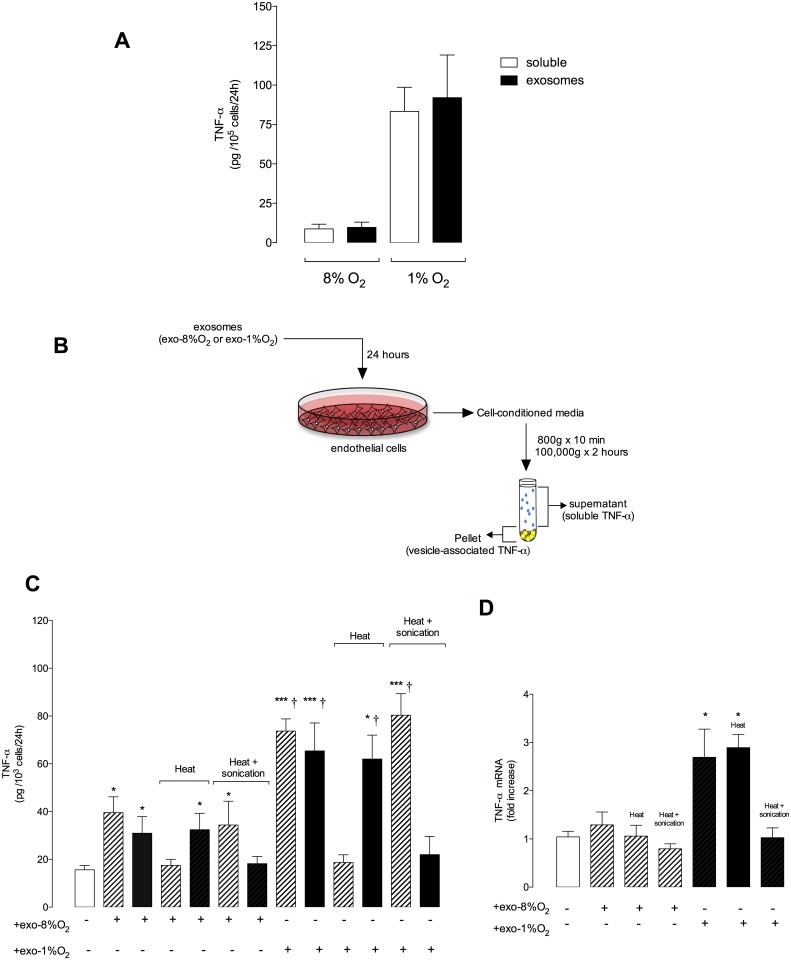
Levels of TNFα in exosome and cell-conditioned media. The concentration of TNFα was quantified in exososomes and EC-consitioned media using an ELISA kit. (A) Exosomes were isolated from EVT cells cultured at 8% and 1% oxygen and the concentration of TNFα was quantified in exosomes and in the soluble fractions (i.e. soluble). The soluble fraction was generated by ultracentrifugation at 100,000g (supernatant). (B) experimental design. (C) EC were cultured in the absence or in the presence of exosomes isolated from EVT culture at 8% or 1% oxygen for 24h and the concentration of TNFα was quantified in EC-derived exosomes and soluble fraction. (D) TNFα mRNA expression. Data represents n = 12 well for each point (6 different experiment in duplicate). Values are mean ± SEM.

The effect of exosomes (100 μg protein/mL equivalent to 5 x 10^8^ vesicles per mL) isolated from EVT cultured under 8% or 1% O_2_ on EC migration and release of TNFα from ECs are presented in Figs 4 and 5, respectively. Exo–EVT cultured under an atmosphere of 8% O_2_, when exposed to HUVEC cells, significantly increased EC migration (*p* < 0.005, n = 6 independent experiments). Exosomes isolated from EVT under hypoxic conditions inhibited EC migration in a time-dependent manner (*p*<0.005, n = 6 independent experiments), as shown by significant differences in the half-maximal stimulatory time (ST_50_) of 5.1 ± 0.28 h compared to 11.9 ± 0.29 h for EC migration under 8% and 1% oxygen, respectively (*p*<0.05, Fig 4A and 4B). The results under the 2 conditions also showed significant differences when compared to the control without exosomes (7.3 ± 0.24 h). The area under curve analysis showed that exosomes isolated from EVT cultured under 8% O_2_ promote EC migration by ~30% compared to the control (without exosomes). Additionally, exosomes from 1% O_2_ significantly decrease EC migration by ~25% compared to the control (Fig 4C).

The concentration of TNFα was quantified in the exosome and soluble fractions (*i*.*e*. 100,000g supernate) isolated from EVT cells cultured under 8% and 1% O_2_. The mean concentration of TNFα was significantly higher in both fractions obtained from cells incubated at 1% O_2_ than when incubated at 8% O_2_ (Fig 5A). Interestingly, exosomes derived from EVT cells cultured under 8% or 1% O_2_ increased (p<0.05) the release of both vesicle-associated and soluble TNFα from EC compared to the control without exosomes (Fig 5B and 5C). The effect of exosomes isolated from EVT under 1% O_2_ on the release of TNFα from EC was significantly greater ~1.8-fold (p<0.05) compared to exosomes isolated from 8% O_2_. Heat treatment of the EC-conditioned media did not significantly affect the concentration of vesicle-associated TNFα. In contrast, sonication + heat completely abolished the concentration of vesicle-associated TNFα in EC-conditioned media. Interestingly, exosomes isolated from EVT under 1% O_2_ increased the expression of TNFα in EC compared with the effect of exosomes from EVT under 8% O_2_ and without exosomes (control) (Fig 5D). Exosomes isolated from EVT cultured under 1% O2 increase ~2.5-fold the TNFα mRNA expression while exosomes from EVT cultured under 8% O2 did not have an effect on the exression of TNFα mRNA.

S3 Fig illustrates a time course of the internalization of EVT-derived exosomes. Exosome uptake by ECs was time-dependent and no significant effect of exosomes isolated from EVT cultured at 8% or 1% O_2_ was identified. Interestingly, heat treatment of exosomes did not affect exosome uptake by ECs. On the other hand, sonication completely abolished the uptake of fluorescent exosomes. The disruption of exosomes by sonication was confirmed by electron microscopy.

### Exosomal miRNA profile in plasma from women at early gestation who develop complications of pregnancy associated with incomplete spiral arterial remodelling

**Fig 6:**
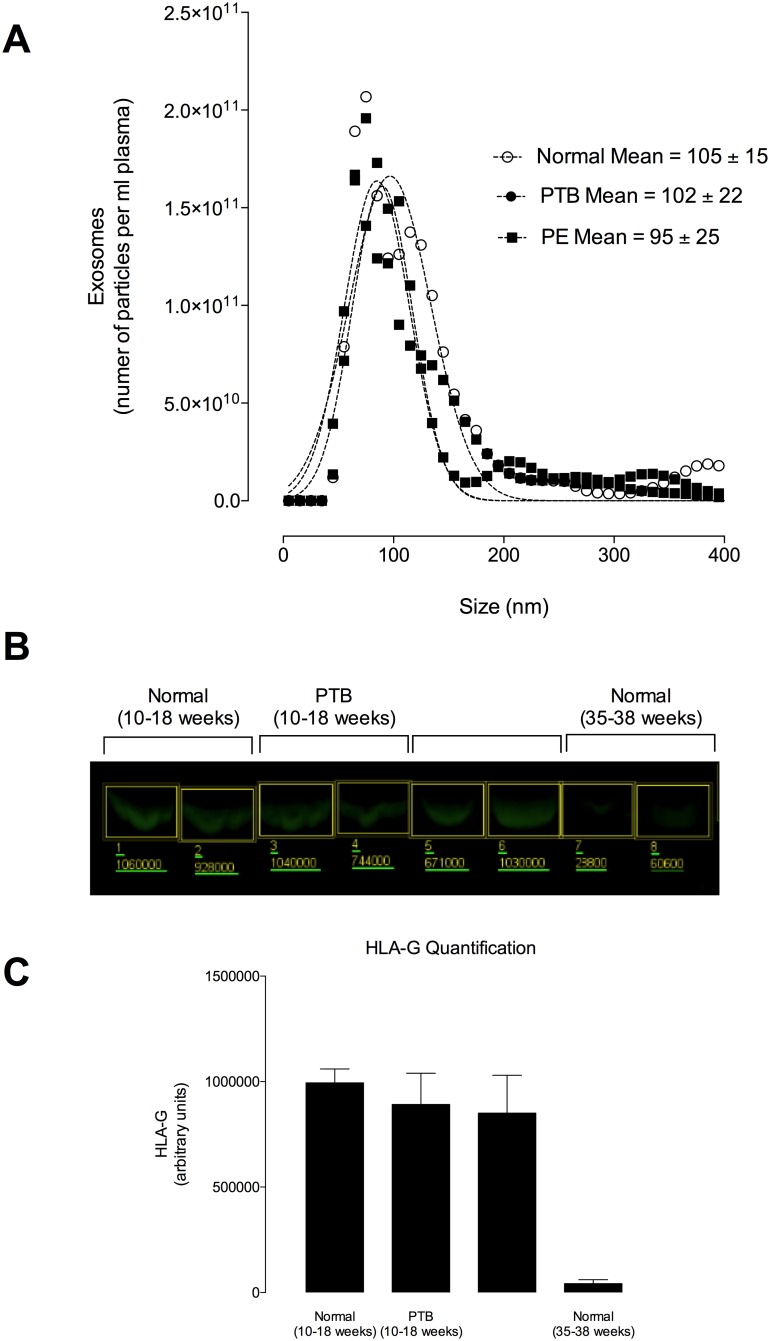
Exosomes present in maternal circulation before 18 weeks are positive for HLA-G. Exosomes were isolated from maternal plasma before 18 weeks of gestation by differential and buoyant density centrifugation. (A) Representative exosomes size distribution isolated from maternal plasma using a NanoSight NS500 instrument. (B) Western blot for HLA-G. (C) Quantification from B.

**Fig 7:**
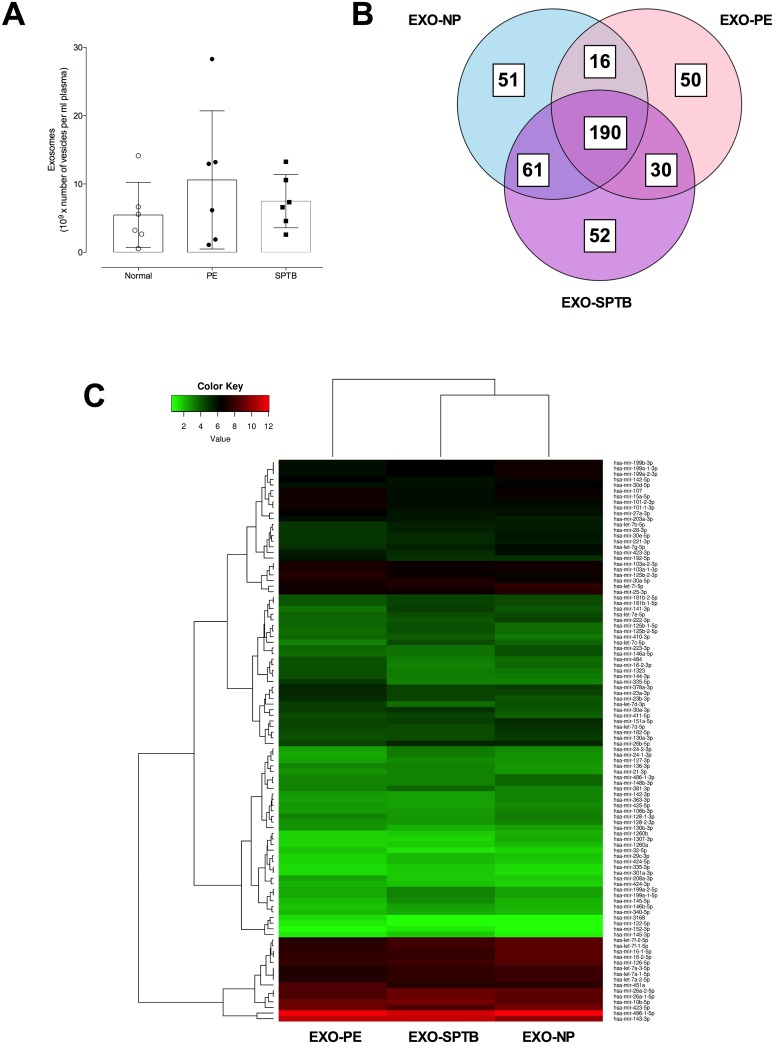
miRNA profile in exosome present in maternal circulation at early gestation. (A) number of exosomes in normal, preeclampsia (PE) and spontaneous preterm birth (SPTB) at early gestation. (B) The Venn diagram shows the distribution of common and unique miRNA identified in exosomes present in maternal circulation in normal (EXO-NP), PE (EXO-PE) and SPTB (EXO-SPTB). (C) Top 100 abundant miRNAs classified by normalised counts (see Methods) identified in exosomes isolated from maternal circulation. Hierarchical clustering of deep sequencing data was performed across exosomal miRNA samples. Normalisation of the reads was performed by DESeq2 using factors derived from the media ratio method.

Samples were collected at early gestation (<18 weeks) and then retrospectively stratified into those who had a normal pregnancy (*i*.*e*. women without chronic medical conditions or obstetric complications), those who developed PE and those who had a preterm birth (PTB). PE is a hypertensive disorder affecting both mother and child, characterized by high blood pressure and protein in urine. The characteristics of exosomes isolated and purified using a well-established and validated method [15–17, 20, 23] are presented in Fig 6. Exosomes isolated from maternal plasma at early gestation were positive for HLA-G and the expression of HLA-G in exosomes present in maternal circulation decreased significantly as the pregnancy progressed (i.e. third trimester) (Fig 6B and 6C). We did not find any significant differences between the number of exosomes across all the conditions (Fig 7A). In women diagnosed with PE and PTB, we obtained 10.59 ± 10.11 x10^9^ and 7.48 ± 3.89 x10^9^ exosomes per mL plasma, respectively. Women with normal pregnancy exhibited 5.4 ± 4.7 x10^9^ exosomes per mL plasma. This was lower compared to PE and PTB, but without statistical difference (ANOVA, p = 0.44).

A total of 450 miRNA were identified in exosomes isolated from maternal plasma. 51, 50 and 52 unique miRNA were identified in exosomes isolated from normal, PE and PTB, respectively (Fig 7B). Hierarchical clustering of deep sequencing data is presented in Fig 7C. This clustering of miRNAs revealed that the majority of the top 100 significant miRNAs have low expression. Furthermore, sample clustering shows that the miRNA profiles from normal and PTB are more similar to one another when compared to PE. Statistical analysis of the common miRNAs identified in exosomes from normal pregnancies and PE pregnancies, and normal and PTB, that were differentially expressed in exosomes samples was conducted by applying a filter of p-value<0.01 and at least a 2-fold change in either direction using Ingenuity pathway analysis. In total 10 miRNAs were down-regulated (miR-335-5p, miR-192-5p, miR-23a-3p, miR-144-3p, miR-125b-2-3p, miR-542-3p, miR-205-5p, miR-208a-3p, miR-518a-3p, and miR-451a) and 10 miRNAs were up-regulated (miR-744-5p, miR-584-5p, let-7a-5p, miR-6724-5p, miR-17-5p, miR-199a-3p, miR-141-3p, miR-30c-5p, miR-26a-5p, and miR-221-3p) in exosomes isolated from PE compared with normal pregnancies. Similarly, in total 5 miRNAs were down-regulated (miR-145-3p, miR-4792, miR-344a-5p, miR-889-3p, and miR-625-5p) and 8 miRNAs were up-regulated (let-7a-5p, miR-17-5p, miR-92a-3p, miR-191-5p, miR-151-3p, miR-423-5p, miR-344d-3p, and miR-32-3p) in exosomes isolated from PTB compared with normal pregnancies.

### Association of miRNA profiles between exosomes isolated from EVT cells and plasma from pregnant women at early gestation

The relationship between exosomal profile of EVT cells and plasma was addressed using a linear regression analysis (S4 Fig). Interestingly, linear regression analysis of common miRNAs identified between exosomes from EVT cultured at 1% (EXO-1%) or 8% (EXO-8%) and plasma from normal (EXO-NP), PE (EXO-PE) or PTB (EXO-PTB) showed a positive association across all comparisons (i.e. EXO-8% or EXO-1% vs EXO-NP, EXO-PE or EXO-PTB). Interestingly, regression analysis showed a strong association between EXO-1% and EXO-PTB, in which changes in the EXO-1% profile accounts for >45% of the observed variation in exosomal miRNA profile in plasma obtained from women at early gestation who develop PTB later during pregnancy (S4 Fig).

**Fig 8:**
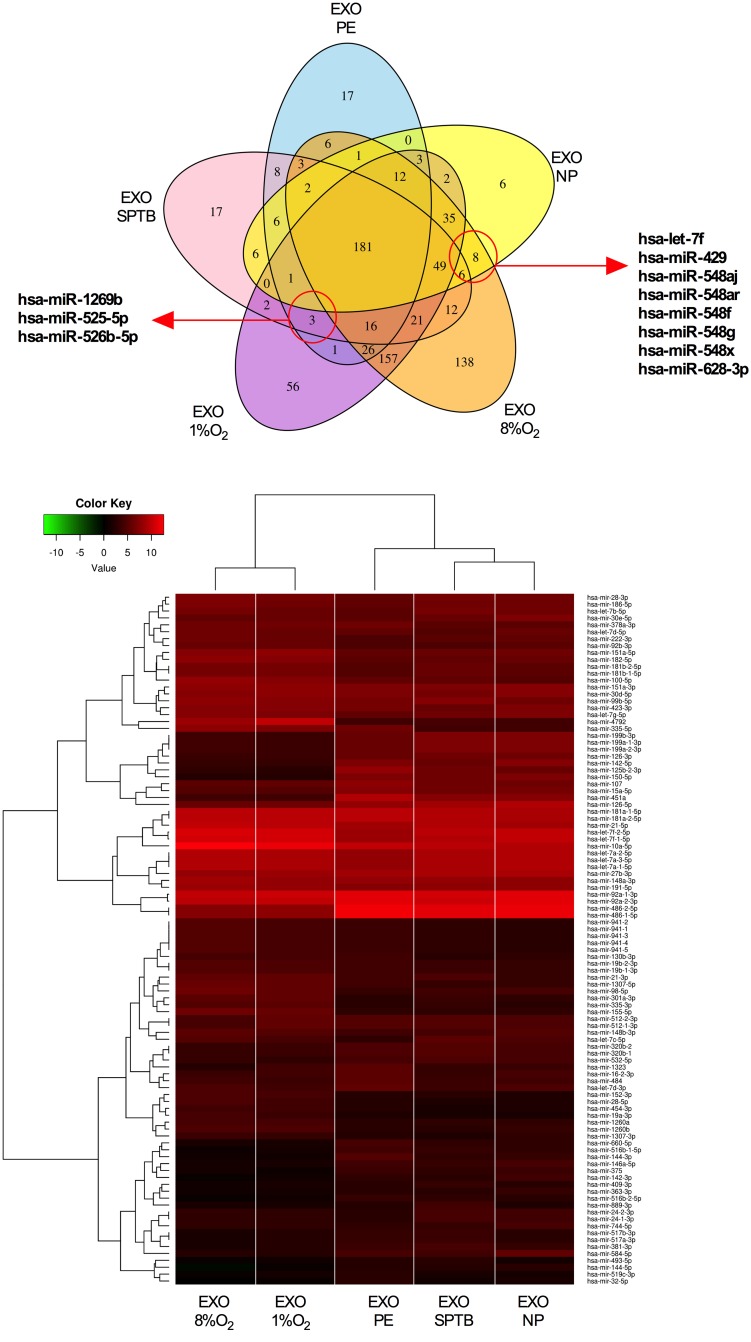
Comparison analysis of miRNA profile between exosomes from EVT and present in maternal circulation at early gestation. (A) Venn diagrams showing unique and common miRNA detected in different exosomes preparations. (B) Highly abundant miRNAs identified in exosomes isolated from EVT cultured under 8% (EXO-8%O_2_) or 1% (EXO-1%O_2_) oxygen and exosomes isolated maternal circulation obtained from women with normal (EXO-NP), Preeclamptic (EXO-PE) or spontaneous preterm birth (EXO-SPTB). Hierarchical clustering of deep sequencing data was performed across exosomal miRNA samples. Normalisation of the reads was performed by DESeq2 using factors derived from the media ratio method.

Additionally, we combined all the miRNAs identified across all samples for exosomes isolated from EVT cells and plasma (Fig 8). Unique and common miRNAs are presented as a Venn diagram (Fig 8A). A total of 181 common miRNAs were identified across all the preparations. Furthermore, exosomes derived from EVT cells contained more unique miRNAs (56 and 138), compared to plasma samples (17, 17 and 6). This observation also correlates with the hierarchical clustering of samples within the heatmap (Fig 8B). Hierarchical clustering of miRNAs identified two large group clusters, showing that the majority of significant miRNAs have high expression.

Finally, 8 miRNAs (hsa-let-7f, hsa-miR-429, hsa-miR-548aj, hsa-miR-548ar, hsa-miR-548f, hsa-miR-548g, hsa-miR-548x, and hsa-miR-628-3p) were identified in exosomes obtained from normal pregnancies at early gestation which were also present in exosomes derived from EVT cultured at 8% oxygen (to mimic physiological conditions). However, these were not present in exosomes from EVT cultured at 1% oxygen (to mimic pathological conditions) or exosomes from maternal plasma from women who later developed PE and SPTB. Similary, 3 miRNA (hsa-miR-1269b, hsa-miR-525-5p and hsa-miR-526b-5p) were identified only in pathological conditions (i.e. present in exosomes from EVT-1% oxygen, exo-PE and exo-SPTB).

## Discussion

Over the course of the last decade, our understanding of how cells communicate with each other under both normal and pathological conditions has been greatly elucidated following the identification of exosomes as key players in cell-to-cell signalling. While our understanding of the molecular mechanisms by which EVT affect vascular remodeling is increasing, there is a paucity of data about how EVTs interact with cells within the uterine spiral artery, including ECs. In particular, the role of EVT-derived exosomes in cell-to-cell communication and vascular remodeling has yet to be considered. This study sought to develop an understanding of the role of exosomes in this key physiological event. The principal findings of this study were firstly that low oxygen tension (*i*.*e*. hypoxia) increased the release of exosomes and modified the exosomal miRNA profile from EVT. It was then found that exosomes from EVT cultured at 1% oxygen decreased EC migration and increased the release of TNFα from EC. Additionally, exosomes released from EVT cells (HLA-G^+ve^) were present in maternal circulation at early gestation. The miRNA profile of exosomes present in maternal circulation from women who later developed PE and SPTB was different compared to normal pregnancies. Therefore, this study is a proof of concept in which we suggest that exosomal miRNA present in maternal plasma at early gestation might be used as a diagnostic biomarker for early detection of complications of pregnancy associated with incomplete SpA remodeling. However, further validation studies using a larger cohort of patients are required.

Remodeling of uterine spiral arteries is crucial for maternal adaptations to pregnancy. Failures in this process may result in pregnancy complications, including PE and fetal growth restriction. While the precise mechanisms involved in uterine spiral artery remodeling have yet to be established, interstitial and perivascular EVT have been implicated. EVT migrate into the maternal decidua and myometrium from 12–16 weeks of gestation in association with vascular remodeling [28]. Furthermore, it has been proposed that inadequate invasion of EVT into the myometrium is associated with a failure to remodel uterine spiral arteries. However, the mechanisms by which EVT interact with the cells of the vessel wall to induce apoptosis and cell migration is unknown.

Recently, we reported that exosomes released from EVT are internalized by vascular smooth muscle cells and subsequently modify their migration capacity [29]. We have also reported that the release of exosomes from cytotrophoblast cells (the progenitor of EVT) is responsive to changes in oxygen tension [15]. Based on these data, we proposed that exosomal signaling is one pathway by which interstitial EVT may induce phenotypic changes in the vascular smooth muscle and ECs of uterine spiral arteries. Furthermore, we suggested exosomal signaling by EVT is oxygen sensitive and that EVT-derived exosomes may facilitate or impede vascular remodeling, depending upon the prevailing oxygen tension. Thus, in this study we hypothesized that oxygen tension regulates the bioactivity and release of exosomes from EVT *in vitro*.

HTR-8/SVneo cells are a commonly accepted model of invasive EVT cells. HTR-8/SVneo cells were developed using first trimester EVT infected with simian virus 40 large T antigen (SV40) [13]. However, numerous studies have investigated wether HTR-8/SVneo cells are a reliable in-vitro model of primary EVT cells. Abou-Kheir *et al*., showed that the HTR-8/SVneo cell line contained a heterogeneous population of trophoblast and mesenchymal/stromal cells [30]. In comparison to other placental cell lines, the expression of trophoblast/epithelial markers such as cytokeratin 7 (CK7) and e-cadherin was silenced in HTR-8/SVneo cells [30–32] while a higher expression of vimentin (a marker of epithelial to mesenchymal transition) was observed [30–33]. Similarly, Takao *et al*., observed a weak expression of HLA-G and integrin alpha-V/beta-3 [34], which are known primary EVT (epithelial) markers [35–37]. Moreover, genome-wide gene expression profiles showed that the molecular signature of HTR8/SVneo cells was vastly different from that of primary EVTs [38]. In addition, the miRNA profile in HTR8/SVneo cells is different compared to primary first and third trophoblast cells and chorioxarcinoma-derived cell lines, mainly because HTR8/SVneo cells did not express C19MC cluster [39, 40]. Therefore, results obtained from HTR-8/SVneo cells must be further verified using the appropriate primary EVT cells.

Exosome bioactivity, as assessed by exosome-induced EC migration and TNFα release, was also oxygen-tension dependent. Exosomes released from EVT incubated under 1% O_2_ induced a 1.8-fold increase in TNFα release and were less effective in promoting cell migration compared to exosomes released from EVT incubated under 8% O_2_. Under normal physiological conditions (*i*.*e*. 8% O_2_), EVT-derived exosomes promote EC migration which is required for the conversion of uterine spiral arteries into high capacity, low resistance vessels. Under low oxygen conditions, however, the ability of EVT-derived exosomes to induce EC migration is repressed.

Our results suggest that oxygen tension regulates the bioactivity of EVT-derived exosomes on ECs, whereby low oxygen tension inhibits EVT migration out of the artery and increases the concentration of TNFα at the site of remodeling. These changes may lead to failures in spiral arterial remodeling and contribute to the aetiology of pregnancy complications associated with insufficient blood supply and placental dysfunction. Interestingly, a dual effect of TNFα in a dose-dependent manner has previously been suggested [41, 42]. TNFα has pleiotropic effects on cell targets, including the activation of cell death and survival pathways. However, the effect of EVT-derived exosomes on EC apoptosis has yet to be established.

### Effect of low oxygen tension on exosome release

Generally, oxygen tensions close to atmospheric conditions (~20%) are used for performing in vitro cultures. Although for some tissues like the human placenta, the physiological conditions are generally lower and are dependent on the gestational age, 10–13% oxygen is the suggested maximum for in vivo oxygen tension. In this study, we cultured EVT cells under 8% and 1% O_2_ to mimic physiological and pathological conditions, respectively. Therefore, as routine cell culture exposes cells and tissues to pO_2_ which exceeds that of blood, even when there is maximum oxygen saturation and the pO2 levels in the placental tissue are lower than those in the blood, further elucidation of how the oxygen tension is sensed by cells can provide insight into variations in cellular mechanisms under different physiological conditions.

Loss of the EC layer is critical to SpA remodeling in which arterial components are replaced by trophoblast cells [3]. Therefore, it is inferred that there is indirect communication between EVT functions and SpA remodeling. The mechanisms by which EVT remodels SpA and how this may be altered under different oxygen tensions, however, have yet to be fully elucidated. We propose that EVT-derived exosomes act as intermediary signaling molecules responsible for communication between EVT cells and the components of the artery, including ECs.

Due to a range of possible communication pathways, insights on how the extracellular conditions of the microenvironment affect cell growth and exosome release are important in understanding the system. Interestingly, placentation at early gestation occurs under low oxygen tension and the oxygen tension increases once the maternal blood flow into the intervillous space is fully established [43]. Recent studies have shown that during a normal pregnancy, exosomal release increases throughout the first trimester from ~6 weeks of pregnancy [20]. Interestingly, the intervillous circulation is not fully established until ~10 weeks of pregnancy, suggesting that the placental exosomes entering maternal circulation before 10 weeks [44] might be mainly EVT-derived exosomes. The data obtained in this study indicates that oxygen tension is an important factor in exosome release from EVT cells. This relationship is similar to that observed in cancer cells, where low oxygen tension is associated with increased exosome release in breast cancer cell lines, [45] and also in malignant brain tumor glioblastoma multiforme (GBM)[46].

The effect of exosomes derived from EVT cells on spiral arterial remodeling has not been previously established. We suggest that under normal conditions, cell-to-cell communication between EVT and the vascular wall of the spiral artery is mediated by exosomes and that exosomal signaling between these cells is oxygen tension dependent. PE has been strongly associated with incomplete SpA remodeling. Accordingly, maternal plasma from women with preeclampsia contained higher concentrations of EVs and exosomes. We suggest that low oxygen tension increases exosome secretion from EVT cells, thus hindering normal SpA remodeling and contributing to the pathophysiology of PE as well as other complications of pregnancy.

### Low oxygen tension regulates the bioactivity of exosomes

In this study, we defined bioactivity of exosomes as the capacity of exosomes to regulate the cytokine release from EC. While aberrant exosome release may be a contributing factor in pregnancy complications, our data suggests that in addition to the number of exosomes, oxygen tension changes the bioactivity of exosomes on EC. These effects might have a role in both normal and pathological conditions.

TNFα release from ECs was higher when cells were cultured with exosomes isolated from EVT cultured under low oxygen tension. TNFα can act as an activator of apoptotic signaling. It can also enhance death signaling of cells when stimulated by detrimental stimuli [47]. In this regard, we suggest that an increase in endothelial production of TNFα levels in response to low oxygen tension-induced release of placental exosomes early in gestation might be a common characteristic of pathological pregnancies (*e*.*g*. preeclampsia). Moreover, previous studies in the cancer field show changes in the exosomal composition in response to low oxygen tensions. For example, exosomes under hypoxic conditions in highly malignant glioblastoma multiforme (GBM) brain tumours show an increase in matrix metalloproteinases, IL-8, PDGFs, and caveolin 1, some of which are already associated with a poor prognosis [46]. Interstingly, exosomes from EVT under 1% O2 increase the TNFα mRNA expression in EC. However, the effect of exosomes were ~4-fold higher in inducing the release of TNFα from EC, while the TNFα mRNA expression was ~2.5-fold higher. These data suggest that the effect of exosomes is induced via the production of TNFα in EC. However, as exosomes also contain TNFα, we cannot disregard the potential transfer of TNFα into EC from exosomes. The mechanism by which exosomes cultured from EVT cells under hypoxic conditons induce the release of TNFα from EC remains to be elucidated, this study suggests that a combination of both transfer of TNFα into ECs and regulation of their mRNA expression via exosomes are involved. Futher studies to analyse the content of exosomes in reponse to different oxygen tensions in EVT cells are required.

Oxygen tension was also shown to affect the miRNA profile of specific populations of exosomes. When exposed to EVT-derived exosomes under either 1% or 8% O_2_, HTR-SV/neo cells produced unique regulatory sequences. A total of 10 miRNAs were up-regulated under hypoxic conditions in exosomes isolated from EVT cultured at 1% O_2_ compared to the normal condition of 8% O2: miR-339-5p, miR-16-5p, miR-10a-5p, miR-935, miR-197-3p, let-7a-5p, miR-324-5p, miR-15b-3p, miR-100-5p, miR-365-3p. Of those upregulated, most are tumor supppressors and are involved in the disruption of cell migration and proliferation. These miRNAs have previously been implicated in cancers, namely miRNA 324-5p in hepatocellular carcinoma, breast cancer, and colon cancer [48–50], miRNA 935 in gastric signet ring cell carcinoma [51], and miRNA 15b-3p in prostate cancer [52]. More specifically, miRNA 16-5p has been found to block the proliferation and blood vessel formation of mesenchymal cells in women with PE [53]. While these miRNA have only been studied for their tumor suppressing effects within various cancers, it is possible that they are also at play here during the disruption of EC migration and proliferation under hypoxic conditions.

A total of 10 miRNAs were down-regulated at 1% O_2_ compared to 8% O_2_: miR-675-5p, miR-1246, miR-293-5p, miR-3178, miR-4792, miR-548o-3p, miR-1273g-3p, miR-762, miR-3182, miR-523-3p. Interestingly, there were tumor suppressors present, such as miRNA 3178 in hepatocellular carcinoma [54] and miRNA 4792 in nasopharyngeal carcinoma [55]. However, there were also regulatory sequences that promote tumor proliferation and migration. For example, miRNA 1246 initiates lung carcinomas [56] and miRNA 762 causes cell proliferation and invasion of breast cancers [57]. This conflicts with previous predictions, as it would be expected that since these downregulated miRNAs promote EC migration and proliferation, they would also be tumour initiatiors. A definitive conclusion for these results based on the contradictory findings from the literature review has yet to be made.

Hypoxia within the placenta results in an increase of EC proliferation to increase the number of capillary loops and terminal villi. This increases the surface area available for contact, thus maximizing oxygen and nutrient transfer [58]. This expansion of the capillary loop also causes an increase in vascular resistance that may compromise feto-placental blood flow [58]. Hypoxic conditions have also been shown to induce placental metabolic reprogramming, where HIF-1 stimulates reductions in oxygen usage, but at the cost of higher glucose consumption [59]. Finding the optimal extracellular conditions for exosome release and bioactivity might enhance cell-to-cell communication within the placental cells and between placental cells and maternal tissues, hence contributing to a normal gestation.

## Conclusions

Successful implantation and early placental development are crucial to successful pregnancy outcomes. When these two factors are compromised, there is a heightened risk of developing pregnancy complications. Ineffective differentiation and invasion of placental cells combined with inadequate remodelling of uterine spiral arteries may disrupt placental perfusion and function. This ultimately compromises fetal growth and development. It has previously been established that EVT cells are found in the uterine spiral arteries. Additionally, the remodeling of these vessels is complete once ECs have been replaced. However, the effects of exosomal signaling by EVTs on the endothelial phenotype has not been previously established. Using HTR8/SV neo cells as a model for EVT, this study demonstrates that low oxygen tension promotes release of exosomes from EVT. These exosomes are bioactive, as they modify the migration capacity and release of TNFα from EC. The oxygen tension within the uteroplacental environment varies according to multiple factors, including gestational age and extent of trophoblast invasion and SpA remodeling. Previous studies have highlighted that trophoblast-derived molecules, such as Fas-ligand and TNF-related apoptosis-inducing ligand (TRAIL), are important during SpA remodeling as they induce apoptosis. Here, we demonstrated that exosomes derived from EVT may play a role in this phenomenon. Therefore, we suggest that regulating exosomal signaling between EVT cells and the uterine blood vessels may ameliorate the impact of placentation defects that are associated with the development of PE, intrauterine growth restriction and/or SPTB. Based on the data obtained in this study, we propose that in complications of pregnancy, such as PE and SPTB, the proinflammatory microenvironment and oxygen tension regulate the release and activity of exosomes derived from EVT. Notably, these exosomes contain various signaling molecules, such as cell adhesion molecules, growth factor receptors, annexins, RAB proteins, and heat shock proteins. During a normal pregnancy, EVT-derived exosomes promote loss of vascular cells facilitating remodeling of the spiral uterine arteries. However, in pathological environments, a hypoxic environment is set up with high concentrations of proinflammatory cytokines (*e*.*g*. TNF-α). This environment may inhibit the facilitatory effect of exosomes on vascular cell migration, thus culminating in inadequate arterial remodeling. As a result, this triggers pregnancy complications Finally, several mechanisms such as migration and apoptosis may contribute to the loss of vascular cells from uterine spiral arteries. These events are not mutually exclusive. Rather, a multifaceted, orchestrated process is required to avoid the loss of vessel integrity and thus potentiate a successful pregnancy. We suggest that trophoblast exosomal signaling may be one of these factors.

## Supporting information

S1 Fig Characterize and authenticate of extravillous trophoblast the cell lines was performed by analysing Short Tandem Repeat (STR) DNA profiles. (TIF)

S2 Fig Exosomes purification. EVT cells were cultured under different oxygen tension (see Methods). Exosomes were isolated from cell-conditioned media by differential and buoyant density centrifugation and enriched using a discontinuous iodixanol gradient containing 40% (w/v), 20% (w/v), 10% (w/v) and 5% (w/v) iodixanol (solutions were made by diluting a stock solution of OptiPrep^™^ (60% (w/v) aqueous iodixanol from Sigma-Aldrich) and centrifuged at 100,000 g for 20 h. Fractions were collected manually from top to the bottom (with increasing density), diluted with PBS and centrifuged at 100,000 g for 2h at 4°C. Vesicles CD63 positive were analysed using Qdot-CD63. (A) Representative Western blot for CD63 of enriched exosomes. (B) Representative vesicle size distribution using a NanoSight NS500 instrument.
(TIF)

S3 Fig Internaliszation of exosomes by EC. A time course of the internalization of EVT-derived exosomes by EC and the intracellular accumulation of fluorescence per cell confluence is presented. No significant effect of oxygen tension under which the exosomes were generated was identified. *Top*: fluoresce images after 24 h. *bottom*: Electron microscope images with and without sonication. Values are mean ± SEM (n = 3).
(TIF)

S4 Fig Association between miRNA profile between exosomes from EVT and plasma. The relationship between the exosomal miRNA profile between exosomes isolated from EVT cells cultured under 8% (EXO-8%O_2_) or 1% oxygen (EXO-1%O_2_); and exosomes from plasma obtained from normal (EXO-NP), preeclampsia (EXO-PE) or spontaneous preterm birth (EXO-SPTB) was evaluated by linear regression analysis. Linear regression analysis between (A) EXO-8%O_2_ and EXO-NP; (B) EXO-1%O_2_ and EXO-NP; (C) EXO-8%O_2_ and EXO-PE; (D) EXO-1%O_2_ and EXO-NP; (E) EXO-8%O_2_ and EXO-SPTB; and (F) EXO-1%O_2_ and EXO-SPTB.
(TIF)
