# Supplementary figures and images for: Oxygen tension regulates the miRNA profile and bioactivity of exosomes released from extravillous trophoblast cells – Liquid biopsies for monitoring complications of pregnancy
Source: PLoS One. 2017 Mar 28;12(3):e0174514. doi: 10.1371/journal.pone.0174514 (PMC5370130; doi:10.1371/journal.pone.0174514)

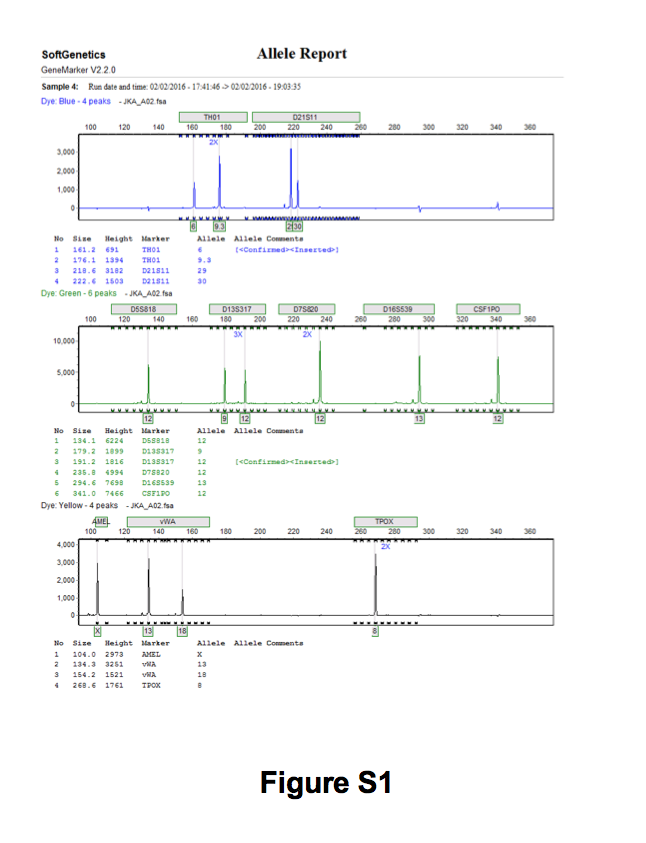

Supplement: S1 Fig — (TIF) [file pone.0174514.s001.tif]

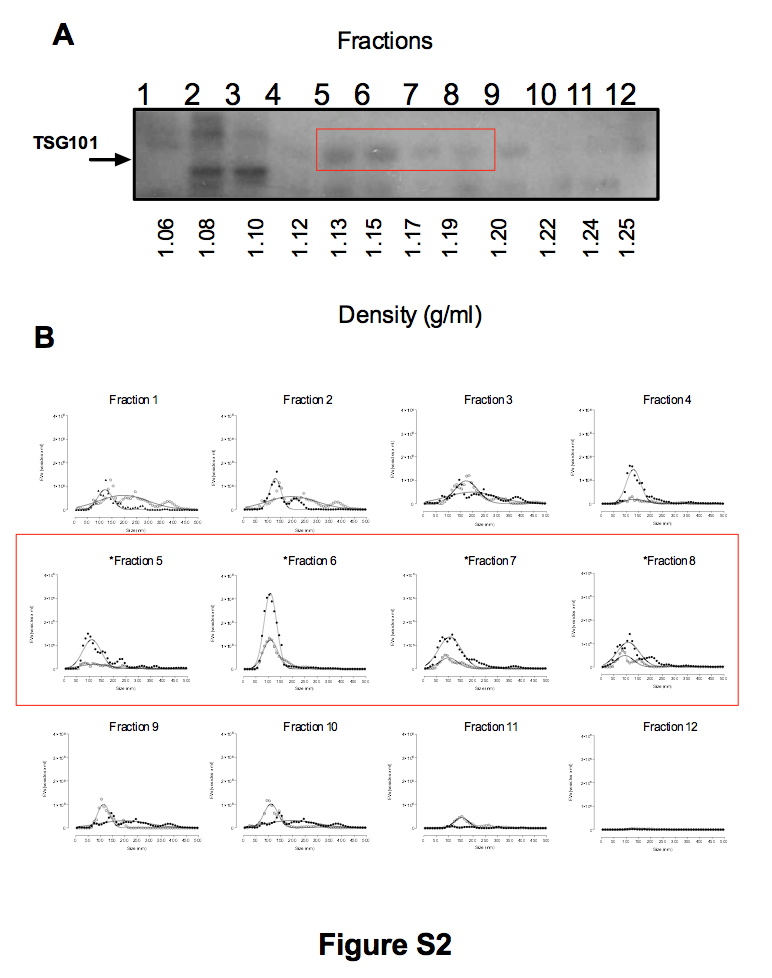

Supplement: S2 Fig — EVT cells were cultured under different oxygen tension (see Methods). Exosomes were isolated from cell-conditioned media by differential and buoyant density centrifugation and enriched using a discontinuous iodixanol gradient containing 40% (w/v), 20% (w/v), 10% (w/v) and 5% (w/v) iodixanol (solutions were made by diluting a stock solution of OptiPrep™ (60% (w/v) aqueous iodixanol from Sigma-Aldrich) and centrifuged at 100,000 g for 20 h. Fractions were collected manually from top to the bottom (with increasing density), diluted with PBS and centrifuged at 100,000 g for 2h at 4°C. Vesicles CD63 positive were analysed using Qdot-CD63. (A) Representative Western blot for CD63 of enriched exosomes. (B) Representative vesicle size distribution using a NanoSight NS500 instrument. (TIF) [file pone.0174514.s002.tif]

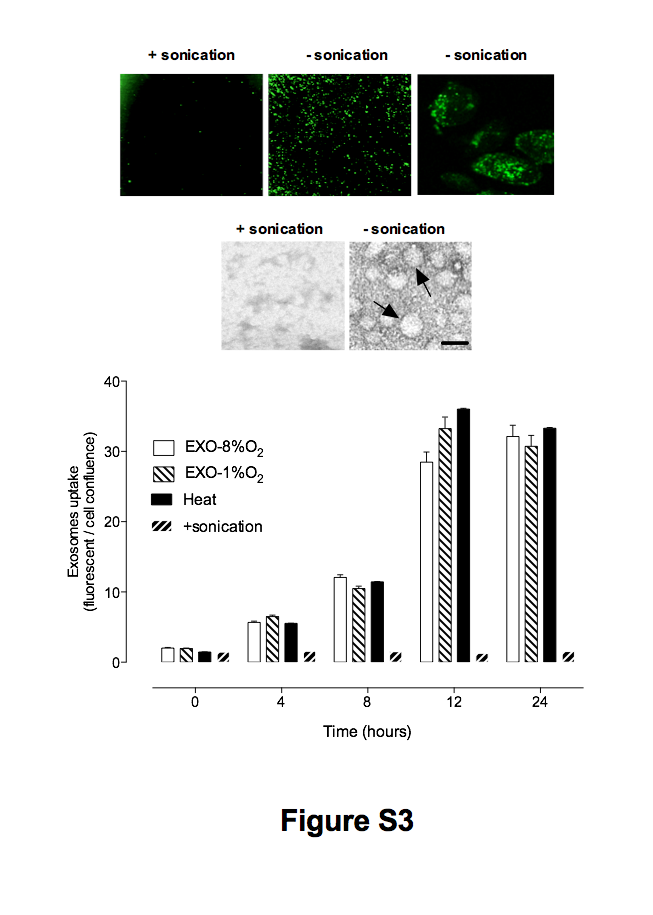

Supplement: S3 Fig — A time course of the internalization of EVT-derived exosomes by EC and the intracellular accumulation of fluorescence per cell confluence is presented. No significant effect of oxygen tension under which the exosomes were generated was identified. Top: fluoresce images after 24 h. bottom: Electron microscope images with and without sonication. Values are mean ± SEM (n = 3). (TIF) [file pone.0174514.s003.tif]

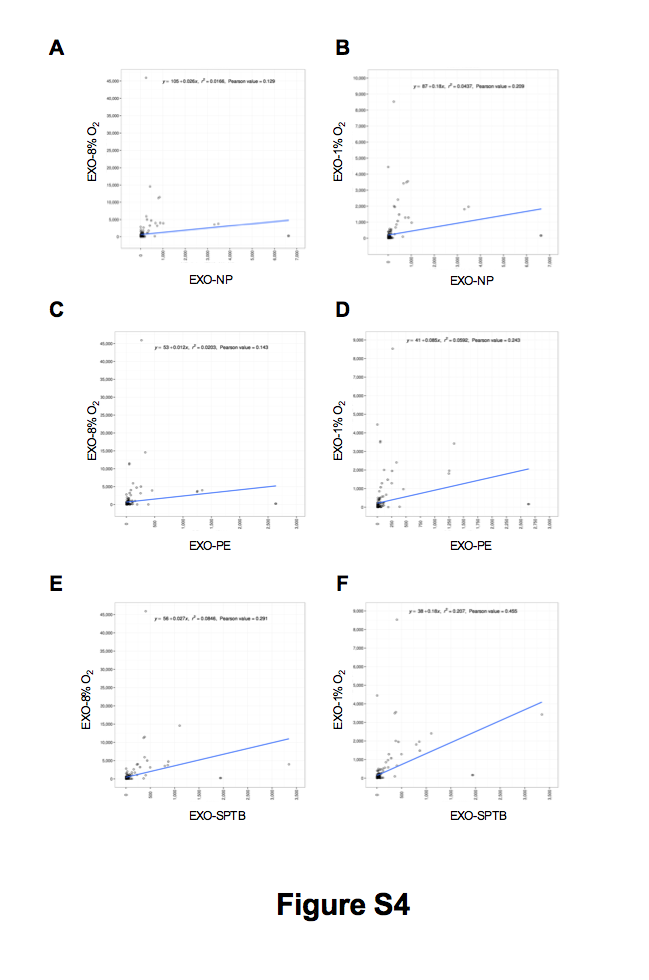

Supplement: S4 Fig — The relationship between the exosomal miRNA profile between exosomes isolated from EVT cells cultured under 8% (EXO-8%O2) or 1% oxygen (EXO-1%O2); and exosomes from plasma obtained from normal (EXO-NP), preeclampsia (EXO-PE) or spontaneous preterm birth (EXO-SPTB) was evaluated by linear regression analysis. Linear regression analysis between (A) EXO-8%O2 and EXO-NP; (B) EXO-1%O2 and EXO-NP; (C) EXO-8%O2 and EXO-PE; (D) EXO-1%O2 and EXO-NP; (E) EXO-8%O2 and EXO-SPTB; and (F) EXO-1%O2 and EXO-SPTB. (TIF) [file pone.0174514.s004.tif]
